# Soluble, Colloidal, and Particulate Iron Across the Hydrothermal Vent Mixing Zones in Broken Spur and Rainbow, Mid-Atlantic Ridge

**DOI:** 10.3389/fmicb.2021.631885

**Published:** 2021-10-29

**Authors:** Mustafa Yücel, Serhat Sevgen, Nadine Le Bris

**Affiliations:** ^1^Institute of Marine Sciences, Middle East Technical University, Mersin, Turkey; ^2^LECOB, SU-CNRS, Sorbonne Université, Banyuls-sur-Mer, France

**Keywords:** hydrothermal vents, iron, redox, nanoparticles, marine ecosystems, deep sea

## Abstract

The slow-spreading Mid-Atlantic Ridge (MAR) forms geological heterogeneity throughout the ridge system by deep crustal faults and their resultant tectonic valleys, which results in the existence of different types of hydrothermal vent fields. Therefore, investigating MAR hydrothermal systems opens a gate to understanding the concentration ranges of ecosystem-limiting metals emanating from compositionally distinct fluids for both near-field chemosynthetic ecosystems and far-field transport into the ocean interiors. Here, we present novel data regarding onboard measured, size-fractionated soluble, colloidal, and particulate iron concentrations from the 2018 R/V L’Atalante – ROV Victor research expedition, during which samples were taken from the mixing zone of black smokers using a ROV-assisted plume sampling. Iron size fractionation (<20, 20–200, and >200nm) data were obtained from onboard sequential filtering, followed by measurement *via* ferrozine assay and spectrophotometric detection at 562nm. Our results showed the persistent presence of a nanoparticulate/colloidal phase (retained within 20–200nm filtrates) even in high-temperature samples. A significant fraction of this phase was retrievable only under treatment with HNO_3_ – a strong acid known to attack and dissolve pyrite nanocrystals. Upon mixing with colder bottom waters and removal of iron in the higher parts of the buoyant plume, the larger size fractions became dominant as the total iron levels decreased, but it was still possible to detect significant (micromolar) levels of nanoparticulate Fe even in samples collected 5m above the orifice in the rising plume. The coolest sample (<10°C) still contained more than 1μM of only nitric acid-leachable nanoparticle/colloidal, at least 200 times higher than a typical Fe concentration in the non-buoyant plume. Our results support previous reports of dissolved Fe in MAR vent plumes, and we propose that this recalcitrant Fe pool – surviving immediate precipitation – contributes to maintaining high hydrothermal iron fluxes to the deep ocean.

## Introduction

Iron redox cycling is intimately coupled with global carbon, phosphorus, and nitrogen cycles. Several transition metals form cofactors of enzymes but also take part in intra- and extra-cellular electron transfer and thus are vital for biology (Twining and Baines, 2013). Especially Fe, Zn, Cu, and Ni were essential in global primary production during certain periods of Earth’s history to the extent that they might have limited global productivity, providing a direct feedback to planetary evolution (Rickaby, 2015; Moore et al., 2017). Moreover, the precipitation of iron as oxides or sulfides (pyrite-FeS_2_) is another major feedback loop in the timing of the atmospheric accumulation of biogenic O_2_. This Fe-initiated feedback also resulted in its massive removal from the oceans since the oxidized form of Fe (Fe^3+^) is insoluble at neutral to alkaline pH if not stabilized in solution by complexation with organics or inorganic ligands, such as sulfides and silicates. Iron is now so scarce in the ocean waters that it limits primary productivity in the so-called “high-nutrient low-chlorophyll” regions, estimated to cover at least one-third of the sunlit ocean (Boyd and Ellwood, 2010). That is why iron fertilization might have driven atmospheric pCO_2_ fluctuations over the glacial-interglacial periods (Jickells et al., 2005), but whether the fertilization originated from external sources (dust input) or internal fluxes (upwelling, vents or seafloor sediments) is an ongoing debate (Winckler et al., 2016).

Since their discovery in 1977 with submersible Alvin, hydrothermal vents have been shown to play a major role in oceanic element cycles. In addition to global impact, hydrothermal vent fluxes also support highly productive chemosynthetic seafloor ecosystems (Le Bris et al., 2019). A major internal flux of iron has also been recognized now, but the poor representation of this source in global models yields uncertainty in the iron cycle feedback to the global carbon cycle (Tagliabue et al., 2010, 2016). When ocean currents support vertical export, the vent-derived iron can influence surface ecosystems. For instance, a recent report by Ardyna et al. (2019) showed how hydrothermal (^3^He enriched) water masses originating from the southern East Pacific Rise can be upwelled along the isopycnals to fertilize phytoplankton blooms in the iron-limited Southern Ocean. That study did not include iron measurements, but recent oceanic transects produced by GEOTRACES (Schlitzer, 2017) showed in all ocean basins how iron can be detected 1000s km away from deep-sea sources (Resing et al., 2015; Fitzsimmons et al., 2017). Such recent findings posed a contrast with the paradigm that Fe rapidly oxidizes and precipitates, leading to its complete scavenging from plumes close to the emission point. Stabilization by organic complexes in far-field (Sander and Koschinsky, 2011; Kleint et al., 2017) has been established as a plausible mechanism; however, a large gap still remains with regard to the issue of metal stabilization near the source. Organic chelators tend to decompose at the temperatures of vent fluids and kinetic considerations point to previously overlooked inorganic geochemical mechanisms involving the formation of nanoparticles in mixing zones (Yücel et al., 2011; Findlay et al., 2015, 2019). Subsequent organic colloid/particle formation away from the mixing zones could further enhance iron export (Toner et al., 2009, 2016). Based on these studies, it is now widely considered that large-scale oceanic transport of iron might be primarily determined by an unknown interplay between inorganic nanoparticles and organic complexes (Gledhill and Buck, 2012; Fitzsimmons et al., 2015, 2017; Buck et al., 2018; Lough et al., 2019a). The known kinetics of “dissolved” ferrous iron oxidation with oxygen (where iron is assumed to exist as Fe^2+^ hydroxy or carbonate species) does not predict such a high stability of iron. Moreover, a complex set of poorly constrained processes occurring at the interface of hydrothermal fluids and deep-sea water, at scales of nanometers to several 10s of meters, seem to determine the export of vent iron to the deep-sea.

Very few works have focused on this spatial scale. While landmark geochemical studies focused on the end-members (Von Damm et al., 1998; Seyfried et al., 2011) and established major processes of water-rock reactions leading to vent fluid chemical composition, chemical oceanography studies focused on 10–1000s of km long water column transects, thus uncovering key aspects of trace element cycling (Wu et al., 2011; Nishioka et al., 2013; Fitzsimmons et al., 2017). Studies on the scales of vent mixing zone interfaces have emerged only recently: one of the first ROV-based rising plume investigations was conducted by Findlay et al. (2015) at MAR (Rainbow, TAG and SnakePit), establishing that a variety of oxides and sulfides is emitted from MAR vents despite the slow-spreading rates and high iron to sulfide (Fe:S>1) ratios in the end-member fluids. Waeles et al. (2017), working in MAR, employed *in situ* filtration (0.45μm) and found that lower Fe:S ratios (<1) may result in iron oxidation processes being more important. This supports the view proposed by Yücel et al. (2011) that pyrite nanoparticles form in high-temperature sulfide-rich hydrothermal solutions, and the formation is negligible at higher dilution ratios as the formation of pyrite is kinetically hindered. Findlay et al. (2019) conducted the first mixing zone profiling with the submersible Alvin in the low Fe:S fluids of the East Pacific Rise (EPR) 9°N and confirmed that if any new pyrite nanoparticles form, the process should be constrained to the immediate vicinity of the orifice. These studies demonstrated kinetic constraints in the mixing zones of hydrothermal vents (as opposed to equilibrium speciation approaches). However, the mechanistic controls behind the formation of the larger colloidal pool in which mineral nanoparticles are situated have remained largely elusive.

In this study, we present onboard measured, size-fractionated dissolved iron results from the 2018 R/V L’Atalante – ROV Victor expedition, during which samples were taken from the vertical mixing zone of black smoker (buoyant) plumes with Titanium majors and a syringe (PEP) multi-sampler operated by the ROV VICTOR 6000. Specifically, we aim to:

- Define the controls on iron size fractionation in different vent structures from Broken Spur and Rainbow in the Mid-Atlantic Ridge, including soluble (sFe), colloidal (cFe), and particulate iron (pFe),
- Explore the dynamics of the colloidal pool with additional measurements of nitric acid-leachable nanoparticle colloids (which we abbreviate as cFe-N),
- Compare two different geological areas, with high Fe:S and low Fe:S ratios in end-member fluids at the Rainbow and Broken Spur vent fields, respectively, for their potential to export iron nanoparticles.

## Study Sites

The Mid-Atlantic Ridge (MAR) is a slow-spreading ridge at a rate of <3cm/year. The geological heterogeneity throughout the MAR system is under the control of tectonic forces (i.e., extreme attenuation of the crust and formation of detachment faults) in relation to magmatic spreading (Schmidt et al., 2007). This special geology of the MAR results in the formation of different types of hydrothermal vents along the entire ridge system (Kelley and Shank, 2010). To this end, Broken Spur and Rainbow hydrothermal vent fields were examined as two important types of vent systems in the MAR.

**Figure 1:**
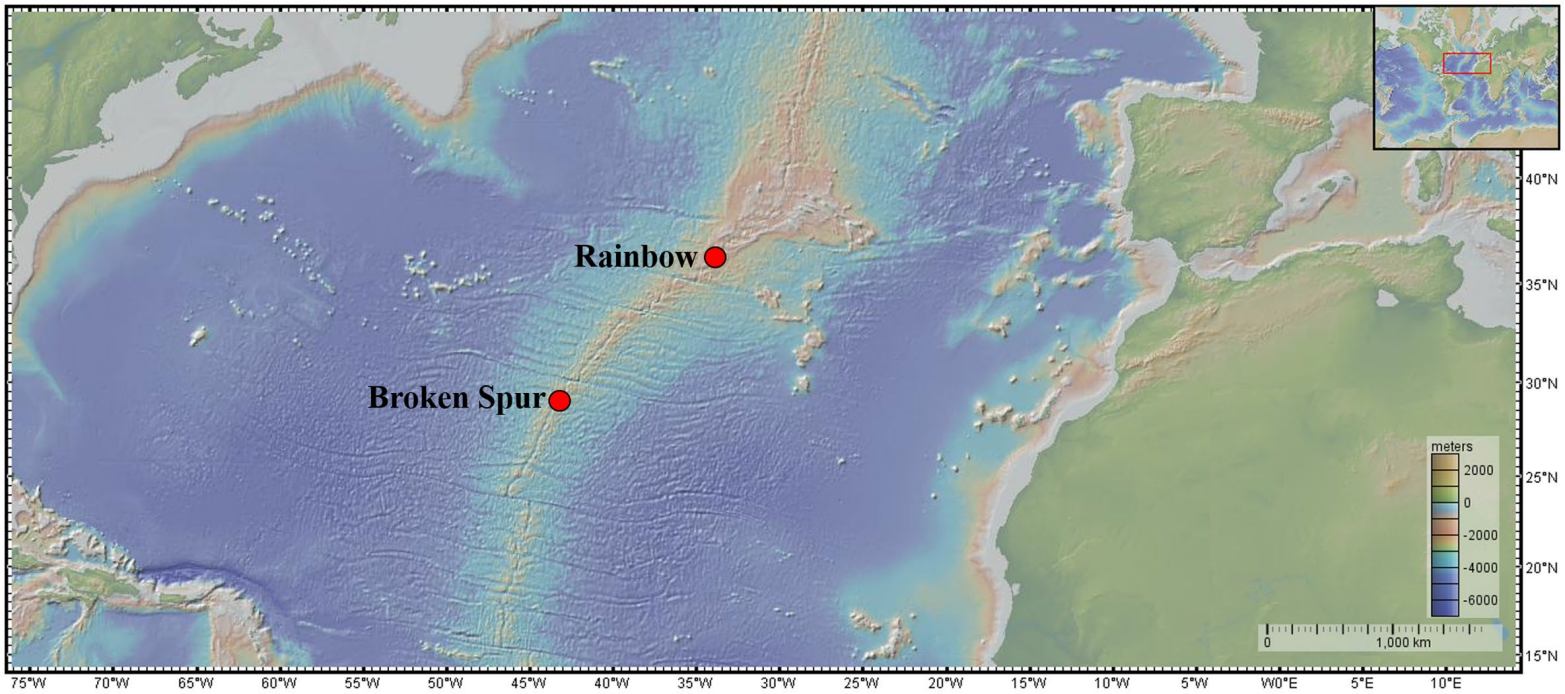
Map showing the location of the vent fields visited along the Mid-Atlantic Ridge. Map courtesy of http://www.geomapapp.org.

The Broken Spur vent field (29° 10' N, 43° 10' W) lies at a depth of ~3,100m on the crest of a neovolcanic zone in the Mid-Atlantic Ridge (Figure 1; Murton et al., 1995), and samples from this field were taken from Spire, Dragon XIII, and Chandelier vent structures. Broken Spur hosts high-temperature black smokers driven by heat extracted from cooling magma. Water-rock reactions with basaltic rocks control the formation and evolution of hydrothermal fluids in the vent field, which has high temperatures (up to 332°C) and low pH vent fluids with a comparable amount of trace metals and a relatively high concentration of sulfides with respect to other vent sites at the MAR (James et al., 1995; Charlou et al., 2010; this study).

The Rainbow vent field is located at 36° 14' N, 33° 54' W at a depth of ~2,300m on a nonvolcanic ridge in the northern MAR (Figure 1; Douville et al., 2002; Charlou et al., 2010). Three individual vent structures (named Fumeur Tres Dense, Fumeur Nouveau, and Magali) were sampled in this vent field. Serpentinized peridotites have the primary role in the evolution of vent fluid geochemistry in the Rainbow field. High-temperature vents (up to 372°C) contain low pH fluids with very high concentrations of Fe and Cl and a relatively low concentration of sulfide with respect to other vents in the MAR (Douville et al., 2002; Charlou et al., 2010; Seyfried et al., 2011; Gartman et al., 2014; Findlay et al., 2015; this work). High concentrations of Cl indicate phase separation in the subsurface and exerts a primary control on metal solubility (Seyfried et al., 2011). Moreover, Rainbow vent fluids show stability in both temporal and spatial scales. Studies carried out in 1997 (Douville et al., 2002), 2008 (Seyfried et al., 2011), and our own study in 2018 show that the fluid composition of Rainbow has not changed substantially over an ~20-year timespan. Samples obtained from individual vents in the Rainbow field exhibit uniform composition, which might indicate that Rainbow fluids might be fed by a single deep source (Charlou et al., 2002; Seyfried et al., 2011).

## Materials and Methods

### Sample Collection and Onboard Measurements of Sulfide and Iron

All hydrothermal fluid samples were collected during the July–August 2018 TRANSECT research cruise to the Mid-Atlantic Ridge with R/V L’Atalante and remotely operated vehicle (ROV) Victor 6000 (Le Bris and The Science Party, 2018). High-temperature hydrothermal fluid samples were collected from individual chimneys in the Rainbow and Broken Spur hydrothermal fields. Fluids were collected using titanium syringes (also called “major samplers”), a PEP sampler equipped with syringe or PE bags and Niskin bottles where appropriate. We successfully collected plume samples from six different chimneys in Rainbow and Broken Spur (3 each). Each plume sampling included a maximum of six discrete sampling points within the buoyant plume, from the high-T end-member to the coolest part (8–12m) of the black smoke. The temperatures of the fluids were monitored with high-temperature probes manipulated by the ROV Victor 6000 just before each sampling. Upon retrieval of the fluid samples at ROV Victor 6000 recovery on deck or using the elevator, onboard sample processing was completed within 1h. Fluid aliquots were extracted for shipboard colorimetric determination of dissolved Fe and dissolved sulfide, while shore-based analysis was carried out for trace metals and major ions.

On board R/V L’Atalante, we measured dissolved free sulfide immediately following sample retrieval *via* the colorimetric methylene blue method with spectrophotometric detection at 670nm (Cline, 1969) with an Ocean Optics spectrophotometer (detection limit 0.5μM). For onboard iron measurements, 10ml of filtered (20 and 200nm pore size) and non-filtered subsamples were treated with 1ml 4N HCl and/or 4N HNO_3_ upon retrieval of the samples (Yücel et al., 2011). HNO_3_ was used to dissolve the (nano)particulate pyrite-Fe present in the samples. This acid treatment lasted 6–10h. All dissolved Fe^3+^ was converted to Fe^2+^ using hydroxylamine hydrochloride as a reducing agent, and the samples were analyzed by the Ferrozine assay for dissolved Fe^2+^ at 562nm wavelength using an Ocean Optics spectrophotometer (Stookey, 1970) with a 1-cm pathlength cell. As a result, the size fractions were named following established conventions: <20nm soluble Fe (sFe), 20–200nm colloidal Fe (cFe), sFe+cFe=dissolved iron (dFe) and >200nm as particulate Fe (pFe). We add to this an operationally defined HNO_3_ extractable fraction minus the HCl extractable iron, abbreviated as cFe-N, as it is composed of colloidal nanoparticulate pyrite and/or non-HCl leachable, but HNO_3_ refers to leachable iron oxides. Iron silicate nanoparticles were not leached this way since stronger acids like HF are needed. The detection limit with the ferrozine assay was 0.5μM, and each of the reported measurements represents an average of two duplicates per sample. The analyses had a precision better than 3%.

### On-Shore Analyses of Major Ions and Metals Other Than Iron

Subsamples for shore-based analysis were transferred to polypropylene tubes to measure non-volatile dissolved species in the vent fluids. Filtered (0.2μm pore size Nylon filters) aliquots were acidified with 1ml 4N HCl immediately after subsampling and were stored at −20°C. The concentration of Mg was determined in ion chromatography (IC) with a Dionex CS12-SC separation column, with methane sulfonic acid eluent and CSRS-I suppressor. Filtration through 0.45μm pore size Nylon filters and 100-fold dilution were applied for each sample before the measurement. On the other hand, the concentrations of Mn and Si were measured by a NexIon 350X Inductively Coupled Plasma Mass Spectrometry (ICP-MS). 1ml 6N HNO_3_ was added, and 200-fold dilution was applied to all subsamples before measurement. Perkin Elmer multi-element calibration standard solution for metals in 5% HNO_3_ with a concentration of 10μg/ml was used to prepare the calibration standards. An internal Yttrium standard was used to correct the intensity deviations during measurement with ICP-MS. Precision for IC and ICP-MS was less than 3%. All concentrations obtained from IC and ICP-MS analysis were calculated using calibration curves for each element after considering dilution factors and molar mass calculations.

## Results

### General Observations From the Sites

This study was conducted in a total of 6 black smokers in Broken Spur and Rainbow (Table 1). At Broken Spur, we sampled a mature (Spire) and two relatively newly forming (named Chandelier and Dragon XIII) chimney structures. Spire is a tall (>10m) and complex edifice, the existence of which was also described by James et al. (1995). The largest hydrothermal flow at the very top of the structure was sampled for this study. Chandelier and Dragon XIII were smaller chimneys with less than 2m in height. As shown in Table 1, Broken Spur chimneys had maximum temperatures of 310–332°C. The samples taken from the highest T (orifice) contained high concentrations of co-occurring dissolved Fe (max 187–744μM) and ∑H_2_S (2.7–5.6mM).

**Table 1:** General sampling information on vent fluids from Broken Spur (BS) and Rainbow (Rbw).

	Lat. (N)	Lon. (W)	Depth (m)	Victor dive	T-max (°C)	dFe at orifice (μM)	∑H_2_S (mM)	Samplings (m above orifice)
BS Spire	29°10' 5.2''	43°10'28.45''	3,060	690	310	188.2	5.6	0, 0.1, 1, 2, 5
BS Chandelier	29°10'3.27''	43°10'26.22''	3,046	692	n.m.	743.6	3.1	0, 0.1, 1, 3
BS Dragon XIII	29°10'2.96''	43°10'27.59''	3,046	692	332	728.7	2.7	0, 0.1, 1.5
Rbw Magali	36°13'75.25''	33°54'22.83''	2,238	694	372	22791.3	bdl	0, 0.1, 1, 2, 3, 5
Rbw M2 (Fum. Nouveau)	36°13'45.18''	33°54'12.78''	2,321	686	320	4978.6	bdl	0, 0.2, 1.5, 4, 7
Rbw M1a (Fum. Tres Dense)	36°13'45.12''	33°54'3.24''	2,238	684	300	5430.7	bdl	0, 0.3, 2, 3, 5, 12

“nm” not measured bdl: below detection limit – T-max are the highest temperatures observed during the particular sampling at the start of the plume profiling. End-member temperatures may differ.

Rainbow hydrothermal chimneys included a “Magali” structure as well as two unnamed structures, tentatively called M1a (Fumeur Tres Dense) and M2 (Fumeur Nouveau). Their maximum temperatures ranged between 300 and 372°C with dissolved iron concentrations reaching to over 22mM. Dissolved free sulfide was below detection level in all high-temperature samples. Detailed end-member geochemistry analyses and ecosystem processes resulting from the 2018 R/V L’Atalante TRANSECT expedition will be given in upcoming papers.

### Size Fractionation in Plumes and Distribution of HNO_3_ Extractable Nanoparticles

**Figure 2:**
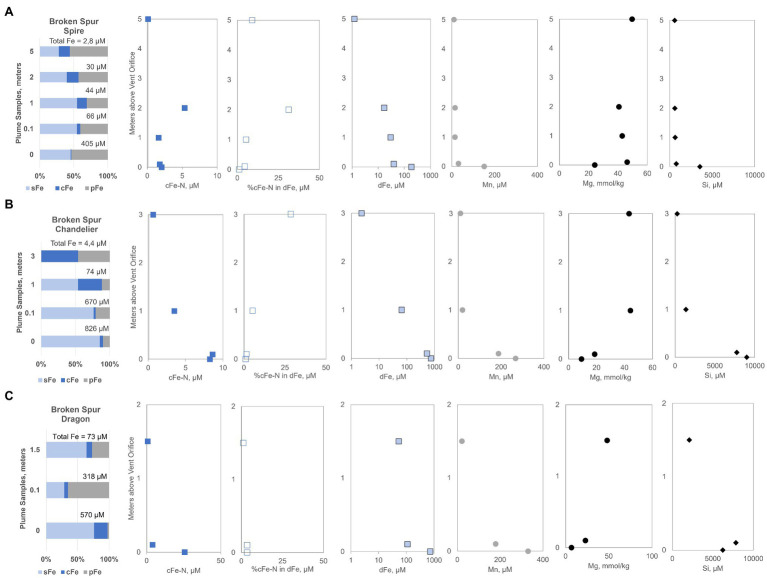
Iron size fractionation data for Broken Spur vent fluids **(A)** Spire, **(B)** Chandelier, and **(C)** Dragon XIII are presented along with profiles containing distance from the orifice vs. measured different chemical species (e.g., cFe-N, dFe, Mn, Mg, and Si). sFe, soluble iron; cFe, colloidal iron; pFe, particulate iron; cFe-N, nanoparticle iron; dFe, dissolved iron.

Spire plume, near the orifice, contained about 405μM of total (TFe=dFe+pFe), which decreased to a TFe of 2.8μM at 5m above the orifice (Figure 2A). Dissolved iron (dFe) decreased from 187.2 to 1.2μM toward the highest sampling point. Colloidal fraction was less than 1% at the orifice but gradually increased to 16% of TFe at 5m. The cFe-N was between 1.8 and 5.4μM, and at 5m, still detectable (0.12μM) levels of cFe-N were present (Figure 2A). Overall, cFe-N was as high as 18% of the TFe in the Spire plume. Compared to Spire, the rising plume of Chandelier was shorter in height, and in the 3-m distance, no detectable sFe was present but more than half of the TFe was present as cFe (Figure 2B). Similar to Spire, the cFe fraction of Chandelier increased with plume height. Dissolved dFe and cFe-N both showed a logarithmic decrease from 743.6 to 2.4 and 8.6 to 0.7μM, respectively. Nanoparticle Fe (cFe-N) comprised about 1% of TFe but rose to 24% of the cFe pool. In the Dragon XIII chimney, the last sampling of Broken Spur, we found that a maximum cFe (21%) fraction was present at the orifice sample, with the pFe fraction peaking in the mid-plume sample (Figure 2C). At 1.5m height, dFe decreased to 53.5μM (from 728.7) and the Fe-nano fraction ranged from 25.6 to 0.6μM. The 25.6μM Fe-nano level is the highest measured in Broken Spur. This is remarkable in the sense that the concentration belongs to the sample obtained from the high-T end. This Fe-nano is about 4% of the dFe emitted from the Dragon XIII high-T fluid.

**Figure 3:**
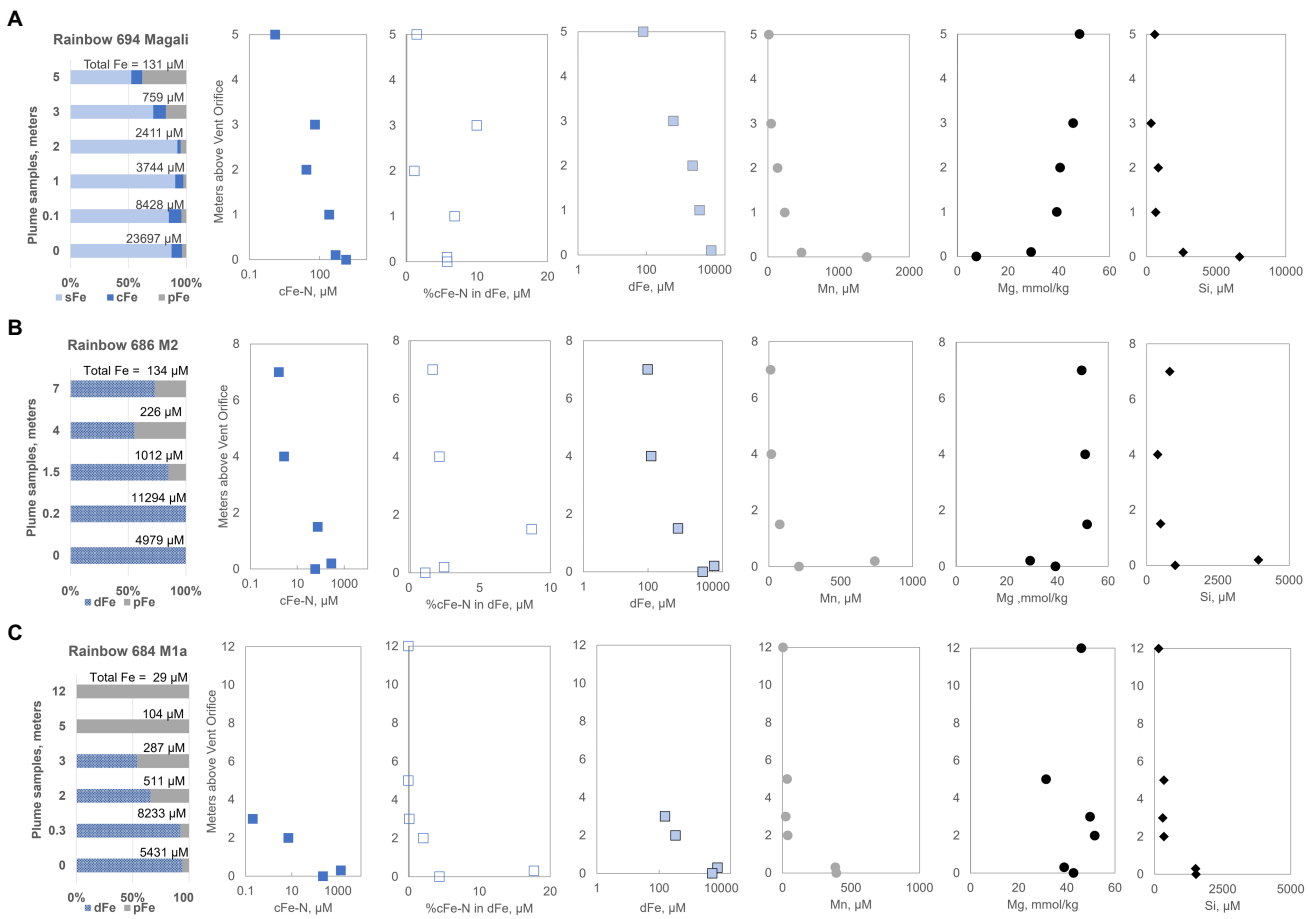
Iron size fractionation data for Rainbow vent fluids **(A)** Magali, **(B)** Fumeur Nouveau (M2), and **(C)** Fumeur Tres Dense (M1a) are presented along with profiles containing distance from the orifice vs. measured different chemical species (e.g., cFe-N, dFe, Mn, Mg, and Si). sFe, soluble iron; cFe, colloidal iron; pFe, particulate iron; cFe-N, nanoparticle iron; dFe, dissolved iron.

Compared to Broken Spur, the Rainbow sites had much higher iron levels at the orifice (Supplementary Table S1). In the Magali plume, about 23,697μM TFe was emitted, and this decreased to TFe of 131μM at 5m (Figure 3A). The sFe fraction was overall high, exceeding 90% in the upper 2m and then decreasing to 52% at 5m. A significant fraction of cFe (max. 11% of the TFe) was present from the bottom to the top of the sampled area, with cFe-N in the cFe ranging from 1326.4μM at the orifice to 1.2μM at 5m (Figure 3A). The cFe-N comprised up to 10% of the dFe pool even 3m above the orifice. The particulate Fe fraction increased with plume height. In the other two chimneys sampled in Rainbow, we could not perform a full-size fractionation including using the 20-nm filter but obtained results on <200 and >200nm filtrates. As was the case with other profiles, the additional Fe released after nitric acid treatment still showed a difference, which we equate to the cFe-N fraction assuming these are colloidal nitric acid-leachable nanoparticles. The M2 plume (Fumeur Nouveau) had 11,294μM TFe at 0.2m but decreased to 134μM at 7m (Figure 3B). Actually, the 0.2-m sample had more iron than our “orifice” sample that was supposed to be richer in iron; however, as can be seen in the Mn, Mg and Si tracer results, the 0.2m sample was much closer to the end-member fluid. Both orifice and 0.2m samples had almost 100% dFe and the particulate fraction became more important at 4 and 7m (Figure 3B). Significant levels (max 281μM) of cFe-N were detected but decreased to 1.6μM at 7m. The cFe-N pool comprised nearly 9% of all dFe at 1.5m in this plume. Similarly, the M1a (Fumeur Tres Dense) plume contained mostly dFe near the orifice with maximum levels of 8,233μM. However, the whole Fe pool in M1a was as pFe at 5 and 12m, with no detectable dFe and cFe-N at less than 5m height (Figure 3C). At 5m, the M1a plume still had a TFe level of 104μM. This is the lowest TFe value when compared to similar heights within the rising plumes of the M2 and Magali hydrothermal vents.

### Plume Vertical Profiles of Mg, Mn, and Si

For a better contextual understanding of the mixing processes in the plume and for assessing the mixing status of the samples, additional dissolved tracer data were obtained. We considered Mg as a conservative tracer of seawater with typical depletion in vent fluids and Mn and Si as fluid tracers as they are also sourced from hydrothermal high-T emissions. In Broken Spur, a maximum of 330μM of dissolved Mn was detected at the orifice of the Dragon XIII chimney (Figure 2C). Other chimneys still emitted Mn levels around 200μM. We observed that in the uppermost 2m about 10μM level of Mn is retained in the plume with little variation. At a height of 5m above the Spire orifice, dissolved Mn was about 9 times higher than dFe (Figure 2A). Mg concentrations displayed expected depletion toward the orifice with a minimum of 6.9mmol/kg measured at Dragon XIII. The Mg profiles of the Dragon XIII and Chandelier plumes display a logarithmic shape implying a regular mixing curve, while the first two samples near the orifice of Spire gave strong indications of a high fraction of seawater mixing just above the orifice (Figure 2). Silica concentrations were high: a maximum of 8,991μM of dissolved Si was found in Chandelier, with decreasing concentrations over the plume height. Several hundred μM of Si was still retained in the upper part of the plume and entered the deep ocean from Broken Spur vents.

Rainbow vents had higher concentrations of Mn (max. 1,398μM) compared to Broken Spur; however, the Mn levels in the upper part of the plumes had a range of 3–12μM, similar to that of Broken Spur (Figure 3). The maximum Si at the high-T end was 6,670μM with a range of 153–807μM. Si was still present beyond the 5-m height. Mirroring the Si profiles, the Magali plume showed a steady decrease with height with a relatively depleted high-T end. M1a and M2 plumes displayed higher levels of seawater mixing, which may also have caused the high fractions of particulates found in these samples.

## Discussion

### A Dynamic Colloidal Pool Controls Size Fractionation and Nanoparticle Dynamics in Plumes

**Figure 4:**
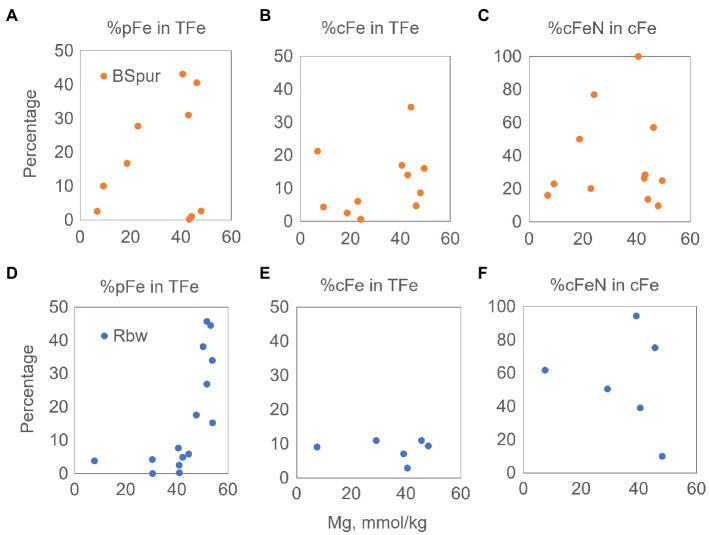
Measured concentration of aqueous Mg versus calculated fractions of %pFe, %cFe in TFe, and %cFe-N in cFe for Broken Spur **(A–C)** and Rainbow **(D–F)** vent fluids. cFe, colloidal iron; pFe, particulate iron; cFe-N, nanoparticle iron; TFe, total iron.

As summarized in Figure 4, the particulate fraction in Broken Spur is significant in all sections of the rising plumes, but in Rainbow, it shows a marked logarithmic increase toward the upper part of the plume. This is possibly related to the lower Fe:S ratios in the Broken spur end-member fluids, which favors the formation of particulates in the high-temperature samples. This would support the findings of Yücel et al. (2011) and Findlay et al. (2015, 2019). In Rainbow, particulate iron formation seems to be largely driven by oxidation in the upper part of the plumes. Although the colloidal fraction is invariant in Rainbow, a slight increasing tendency occurs in Broken Spur. With an average of 15%, the Broken Spur colloidal fraction exceeds that of Rainbow (8%). While quantitatively Rainbow’s input is more significant, Broken Spur’s lower Fe:S ratios might have favored colloidal iron formation. The colloidal fraction of iron in the Broken Spur proximal vent plumes, presumably made up of mostly sulfides, could be produced in the subsurface or very near the orifice as shown by Yücel et al. (2011) and Findlay et al. (2019) for similar low Fe:S EPR vent fluid and plumes. The colloidal fraction grows toward the upper part of the rising plume as the vent fluid continues to mix with deep-sea water. In Rainbow, oxidative processes probably contribute to particulate formation, but this is not reflected in the colloidal fraction. Two hypotheses can be proposed: 1- a stable colloidal phase is formed early on and transported conservatively upwards, where Fe oxides are quickly transformed into large particles and settle 2- the initial colloidal phases (which form in the high-T fluid) are lost but new phases that form in the plume enter the colloidal phase, implying a dynamic colloidal pool.

For further insights into the composition of the colloidal pool, we also investigated the relationship between the fraction of nitric acid-leachable colloids (cFe-N – which we attribute to Fe mineral nanoparticles except for refractory silicates) in the whole cFe pool mixing with seawater. As shown in Figures 4C,F, the range of cFe-N in both sites can be as much as 100% of the whole cFe, and the two vent fields do not show a significant difference in that regard. This maximum is reached around the 40mMMg level, which corresponds to the first 1–1.5m above the orifice, showing that the cFe-N pool is produced below and immediately above the vent orifice. The upper parts of the mixing plume are more labile; hence, HCl extractable colloids become more important. In Broken Spur, this could be due to FeS nanoparticle/cluster formation, which would be retrievable through a mild acid treatment. In Rainbow, the relative stability of the cFe fraction was mentioned earlier, but here, we find that its composition may shift to more labile, plume-forming components. This point then may support the second hypothesis above, implying a dynamic pool of colloids in Rainbow perhaps supplanted by the mixing-induced formation of new phases. Even so, it must be noted that each site at the top of the sampled area emits significant (max 0.7μM in Broken Spur and 1.6μM in Rainbow) levels of cFe-N, emphasizing the early production and conservative mixing of possible nanoparticles of pyrite, non-labile iron oxides (goethite, hematite) or more elusive phases of HNO_3_ extractable silicates and even organic Fe phases.

### Interplay of Silicates, Sulfur, Oxygen, and Iron in Controlling (Nano)Particle Composition

The Rainbow and Broken Spur vent fields provide insight into not only on understanding how much cFe-N is being produced in different vent settings but also how different geochemistry may affect the compositional evolution of (nano)particles both from the subseafloor and throughout the rising plume. It has already been shown that Broken Spur has fluids rich in ∑H_2_S and a comparable amount of Fe, which creates low Fe:S ratios. On the other hand, Rainbow produces fluids with much higher Fe:S ratios (Douville et al., 2002; Charlou et al., 2010). It is also important to notice that the relatively high concentrations of other transition metals, such as Cu and Zn (e.g., Cu and Zn: ~260μM from Seyfried et al., 2011), observed in Rainbow fluids have significant implications on sulfide dynamics in the Rainbow hydrothermal mixing zone since they can precipitate with sulfide species at decreasing temperatures and remove a comparable amount of sulfide from the Rainbow fluids (Findlay et al., 2015).

The distinct geochemical systematics characterizing both vent fields have substantial implications on the quantitative production of cFe-N. For example, one of the main reasons behind the much higher concentration of cFe-N just next to the orifice and throughout the rising plume in the Rainbow vents might be that the Rainbow fluids have a much higher end-member Fe concentration (24–26.5mM) compared to Broken Spur (570–900μM). Around 0–0.1m from the orifice, cFe-N concentrations were between 1,326–467μM in Magali, 224–1,346μM in Fumeur Tres Dense, and 57–281μM in Fumeur Nouveau (Figures 3A,C). On the other hand, quantitatively a much smaller cFe-N pool was detected at the immediate exit of the Broken Spur fluids, reaching only 2–1.8μM in Spire, 8.24–8.61μM in Chandelier and 25.6–3.9μM in Dragon XIII (Figures 2A,C). Despite the large initial Fe difference at the high-T end of the plume, the cFe-N does not differ significantly in the upper parts of the buoyant plume in the Rainbow fluids when compared to Broken Spur. For example, we have measured 1.23, 1.64, and 0.2μM of cFe-N in the coolest hydrothermal fluid samples from the Magali, Fumeur Nouveau and Fumeur Tres Dense orifices, respectively. On the other hand, cFe-N was 0.1μM at 5m from Spire orifice, 0.67μM at 3m from the Chandelier orifice, and an even sharper decrease in Dragon XIII, where cFe-N was measured as 0.62 at 1.5m from the orifice. It seems, therefore, that although the Rainbow fluids create slightly more cFe-N at the immediate exit from the orifice, a higher fraction of the cFe-N pool is being lost in the rising Rainbow plumes, while the cFe-N in the Broken Spur fluids seems more conservative.

**Figure 5:**
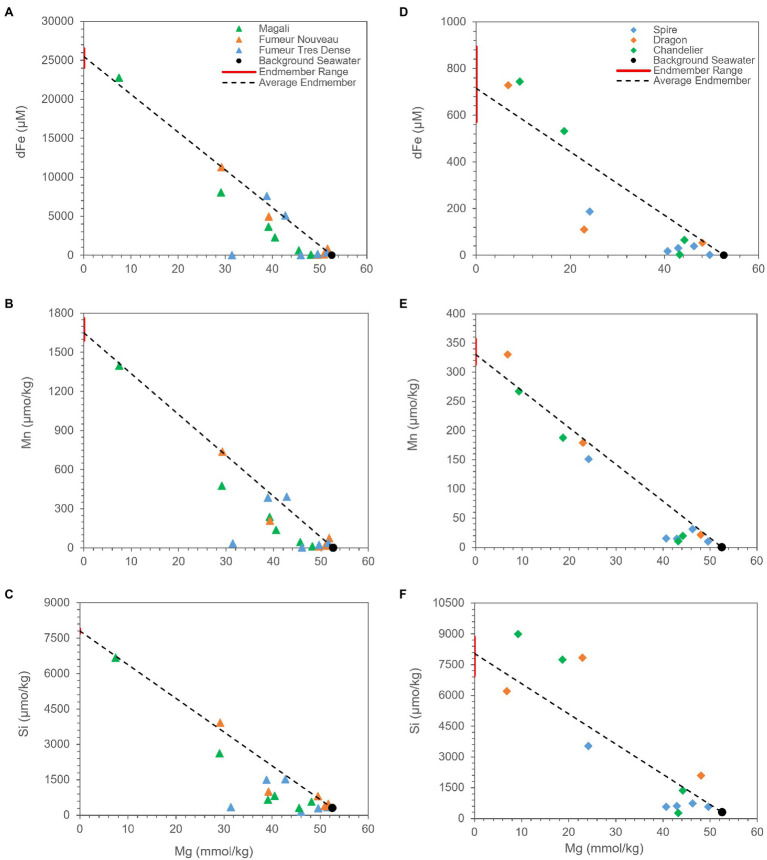
Measured concentrations of aqueous Mg vs. dFe, Mn, and Si for fluid samples from Rainbow **(A–C)** and Broken Spur **(D–F)** vents. For each species, the range in extrapolated zero Mg high-temperature end-member composition is shown with the red bar along the y axis, and the average is shown with a black line projected from seawater composition. Species that exhibit conservative behavior during mixing plot on the line, while species that behave non-conservatively plot above or below the line.

In order to understand the plume dilution and evolution mechanisms creating this shift in the rising plume, correlations for Fe, Si, and Mn were examined for each of the vent fluids. Additionally, since Mg exhibits a conservative behavior during the mixing of end-member hydrothermal fluids with seawater, we preferred to use Mg as a mixing tracer (Figure 5). Seawater is rich in Mg, while hydrothermal fluid end-members have near-zero Mg concentrations due to the reactions of fluids with basalt, gabbro, and peridotite at high temperatures and low water/rock ratios (Bischoff and Dickson, 1975; Mottl and Holland, 1978; Seyfried and Bischoff, 1981; Janecky and Seyfried, 1986; Seyfried et al., 2007). Therefore, a mixing line is created between the average end-member fluid compositions of corresponding vent fluids and background seawater (e.g., McDermott et al., 2020). Comparison of element distributions with the mixing line indicates removal and/or enrichment of corresponding element.

**Figure 6:**
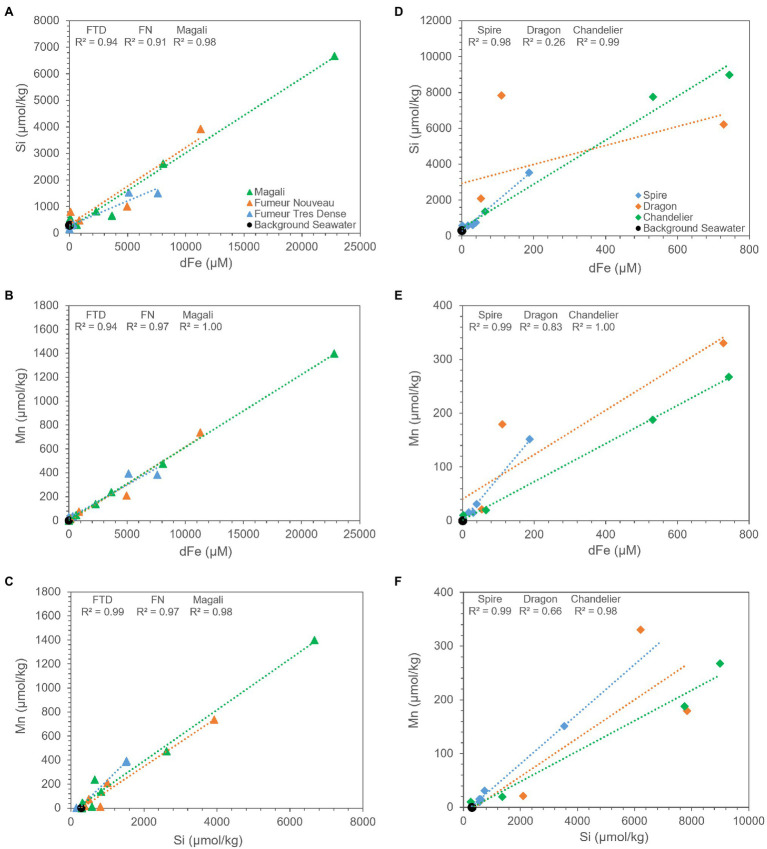
Relationship between measured concentrations of dissolved iron, Si, and Mn in Rainbow **(A–C)** and Broken Spur **(D–F)** vents. Rainbow fluids exhibit very high correlation with Si and Mn in all its vents likewise Broken Spur vent fluids except for Dragon XIII, where scarcity in data might create deviation from correlation for both Si and Mn. Statistical parameters are given on the plots.

Dissolved iron in the Rainbow and Broken Spur fluids exhibits strong positive correlation with Si and Mn (Figures 6A,D), except for the Dragon XIII fluids. These strong correlations show that Si is an important cue to iron and manganese geochemistry in both Rainbow and Broken Spur fluids, and its geochemical behavior in the plume might resemble that of Fe and Mn. The strong positive correlation between Si and Fe and the similar removal trends they exhibit for some sites might support the hypothesis that Fe and Si precipitate together (or aggregate as nanoparticles) in the rising plume. As has been shown, silicates impact Fe (II) oxidation rates (Jones et al., 2009; Kinsela et al., 2016) and Fe (III) silicate complexation could be critical under high silicate concentrations (Patten and Byrne, 2017). Therefore, Fe (II) silica co-precipitates may play a role in stabilizing colloids (Mayer and Jarrell, 1996; Francisco et al., 2020). Findlay et al. (2015) indicated that a substantial amount of Fe (in the mM level) would stay in the buoyant plume and might precipitate in and/or be adsorbed by silicate forms. Different studies have shown that Si-containing particles, such as amorphous silica and kaolinite, are present in hydrothermal vent plumes (Feely et al., 1990; Gartman et al., 2014). However, silicate phases might not be restricted to only these forms, and Fe containing silicate (nano)particles have been shown to be an important fraction in plume geochemistry (Gartman et al., 2014; Estes et al., 2018).

Indeed, the existence of iron silicate nanoparticles is evident in Earth history, and recent studies have suggested that they (e.g., stilpnomelane and greenalite) might have been the primary sediments mediating mobilization and transportation of iron in the Precambrian oceans (Rasmussen et al., 2015; Johnson et al., 2018). Furthermore, the oxidation rate of iron in hydrothermal plumes in deep Atlantic waters is very high compared to other basins (e.g., oxidation half-lives of iron are ~17 and ~27min for Rainbow and TAG plumes at the MAR, respectively) (Field and Sherrell, 2000; Gartman and Findlay, 2020). Different studies have indicated that the oxidation products of dissolved iron (Fe-Ox) are crucial players in plume geochemistry since they form a considerable fraction of the Fe pool and act as strong scavengers for different elements (Mottl and McConachy, 1990; Rudnicki and Elderfield, 1993; Feely et al., 1996; Field and Sherrell, 2000; Breier et al., 2012; Fitzsimmons et al., 2017; Waeles et al., 2017; Hoffman et al., 2018). Therefore, in addition to Fe-Si forms, Fe-Ox might be responsible for sustaining a considerable amount of dissolved iron in the rising plume as well.

Mn is assumed to exhibit conservative behavior during plume development since its oxidation rate is very slow, and it does not tend to precipitate in sulfide phases (Feely et al., 1994; Breier et al., 2012; Resing et al., 2015). However, our data demonstrate that Rainbow and Broken Spur fluids show depletions with respect to Mg (Figures 5B,E). In Rainbow fluids, Findlay et al. (2015) observed a similar Mn removal trend within the first 1-m from the vent orifice. It has already been shown that Mn removal rates can differ from basin to basin. Moreover, different processes, such as the microbial oxidation of Mn and the scavenging of Mn with particles like Fe-Ox, might be responsible for Mn removal in the plume (Campbell et al., 1988; Cowen et al., 1990; Dick and Tebo, 2010; Dick et al., 2013). Additionally, Findlay et al. (2015) indicated that Rainbow has much more particulate Mn when compared to other low Fe:S vent fluids, such as TAG. Since Mn oxidation is not expected within the first couple of meters from the orifice, a major portion of particulate Mn might come from the incorporation of Mn into silicate phases and/or the scavenging of Mn by other particles. A strong positive correlation between Mn and Si can be seen in Figure 6C, and their similar removal trends are shown in Figure 5, which might support the idea that Mn and Si are closely related to each other in the rising plume. As a result, the relatively higher Mn removal trend starting at heights shallower than the orifice in Rainbow fluids might indicate that oxides and silicates have pivotal roles in the compositional evolution of Rainbow plumes when compared to Broken Spur, where Mn removal exhibits relatively higher conservative behavior (Figures 5B,E).

Various studies have indicated that sulfide species are responsible for partitioning Fe into mainly sulfide forms in vent fluids (Mottl and McConachy, 1990; Field and Sherrell, 2000). The main controlling mechanism in the formation of Fe bearing sulfide (nano)particles is attributed the Fe:S ratio of the hydrothermal fluids (Field and Sherrell, 2000). For example, German et al. (2002) calculated that precipitation of iron with sulfides accounts for 80–90% of dissolved iron removal in low Fe:S 13°N EPR fluids, while the formation of Fe-Ox particles is responsible for 10–20% of dissolved iron removal. However, Edmonds and German (2004) indicated that the high Fe:S ratio of Rainbow fluids causes only 4% of total Fe precipitating as sulfide minerals. A more recent study conducted by Waeles et al. (2017) showed that a relatively smaller fraction of hydrothermal Fe (<10%) is precipitated as sulfide minerals, and Fe oxidation is a major process in the removal of hydrothermal iron in vent systems. Nevertheless, different studies agree that Fe-sulfide and Fe-Ox phases (as well as mixed oxide/sulfide phases) are essential parts of the plume geochemistry in varying proportions, depending on the setting.

As an additional point, Figures 2, 3 indicate that Fe fractions (both dFe and cFe-N) exhibit an apparent exponential loss as the hydrothermal plumes rise. Therefore, we examined the e-folding loss rates of dFe and cFe-N in the Rainbow and Broken Spur plumes in order to better estimate the removal trend of these species (Supplementary Figures S1, S2). This analysis shows that simple dilution is not the only controlling mechanism in the removal of Fe phases, as also indicated in Figure 5. On the other hand, the removals are all related to the first order dependence of dFe and cFe-N concentrations. Furthermore, the slopes of the trend lines in Supplementary Figures S1, S2 might give clues about removal rates in the rising plumes. For example, Broken Spur has a higher dissolved iron removal rate (~%33) when compared to the Rainbow rising plume. Accordingly, earlier domination of particulate iron in the Broken Spur plume is valid, as indicated in Figure 4. Additionally, the removal rate of cFe-N phases in the Broken Spur plume is much less when compared to dFe. It indicates that stable FeS (nano)particles exhibit more chemical stability. However, the presence of more labile (nano)particles (e.g., oxides) in the Rainbow rising plume decreases the overall stability of cFe-N and increases the rate of removal as the plume evolves.

Our results suggest that Fe-Si and Fe-Ox (nano)particle forms might become a critical fraction of the colloidal pool during fluid ascension and dilution for both vent fields. At Broken Spur, however, a significant fraction of sulfide nanoparticles might exert the primary control on compositional evolution cFe-N in the rising plume. We also suggest that Rainbow fluids have a much more dynamic environment in the case of cFe-N formation as a result of silicates and oxides forming in the plume, while the immediate formation and predominance of sulfide forms in Broken Spur fluids generate more stable forms of cFe-N. Therefore, a dynamic colloidal pool exists at Rainbow and might be responsible for the observation of a shift of a very high concentration of cFe-N at the vent orifice but the removal of a large fraction up in the buoyant plume. Additionally, metal silicate phases are not thought to be extractable with HCl and HNO_3_ treatments (Huerta-Diaz and Morse, 1992); therefore, quantitative understanding of the cFe-N silicate pool in the rising plume is required for future studies. As an additional factor, organic matter should not be entirely disregarded in the high-T mixing zones. Entrainment near and around diffuse and high-T vent orifices, and the stabilization of nanoparticles with OM before the OM is thermally degraded also needs to be taken into account in the near-field export of iron. Hence, in addition to the now better understood role of inorganic sulfide nanoparticles, the complex interplay of silicate phases as well as the organic derivatives from local chemosynthetic-sourced organic matter should be accounted for (Bailly-Bechet et al., 2008; Bennett et al., 2011).

### Input of Fe to the Deep Atlantic and Its Role in Biogeochemical Cycles

The lowest temperature samples in our mixing zone profiling (<10°C) still contained at least 0.5μM of only nitric acid-leachable nanoparticle/colloidal iron, which is approximately 200 times higher than a typical Fe concentration in the non-buoyant plume. The larger colloidal pool (cFe) reached a maximum of 12μM in the upper part of the mixing zone, constituting a significant flux of cFe to the deep Atlantic. Kunde et al. (2019) reported full water column depth profiles in the Northern Atlantic and found that up to 76% of TFe was cFe in the deep-sea part of the profile and cFe rose to 92% in the samples from a hydrothermal plume, where concentrations increased to as much as 30nM. They proposed an hourglass shape for Fe vertical distribution in the Northern Atlantic, where cFe becomes the dominant Fe phase in the euphotic zone and deep zone. This is consistent with earlier work by Fitzsimmons et al. (2015), which claims an 89–96 cFe percentage in the plume of the nearby TAG vent field. While it is still unclear whether the cFe detected is fully hydrothermal in origin or represents cFe recycled from sFe and pFe from the vents, it is at least possible to achieve these levels from a simple dilution of a high-T produced colloidal pool, including HNO_3_ extractable pyrite-type nanominerals. While Findlay et al. (2015) showed the emanation of Fe, Zn, and Cu sulfides from the MAR, solid state particulate speciation work from the upper part of the plume found no evidence of Fe-sulfide particles in the neutrally buoyant plume of TAG (Ohnemus and Lam, 2015). Lough et al. (2019b) also failed to detect any iron sulfides in the water column in the slow-spreading Von Damm vent field in the Caribbean Sea. Such high Fe:S vent fluids might result in low levels of dissolved sulfide. Accordingly, the slow-spreading systems examined so far perhaps emit limited levels of pyrite nanoparticles, but our results show that they may still be forming in high-T end-members. Silicate-including nanoparticles may also have a dominant role, especially in Rainbow-like systems (Estes et al., 2018; Luther et al., 2020). It can be further proposed that such particles may offer substrates for plume microbes using sulfur or iron as electron acceptors, thus leading to the redissolution and precipitation of iron under different oxide and organic phases.

The extrapolation of our results to broader lengths of the MAR system as well as to the global ridge system is less straightforward, but it can be stated that the spreading rate can exert a first order control on stable iron fluxes. In particular, Rainbow could be representative of serpentinized systems that may be widespread on slow/ultraslow-spreading ridges with a potential for high Fe:S end-members. Previously, based on low ^3^He emissions and their specific topography, slow-spreading systems were thought to export less Fe to deep ocean basins. Now, at least for slow-spreading MAR, it is becoming established that a persistent colloidal pool is generated due to hydrothermal emissions, and iron nanoparticles may play a role in generating a high flux in the early phase of plume formation. While our digestion scheme did not include silicate fractions, our results on Si, Mg, and Fe collectively show that silicate nanoparticles can play a larger role in this type of slow-spreading system. More work integrating a comparative, multi-site sampling will possibly reveal different ridge or back-arc types generating a specific carrier phase, eventually providing different and improved parametrization possibilities for models. Both deep-sea ecosystem models and carbon cycling (German et al., 2015; Le Bris et al., 2019), as well as global climate models (Tagliabue et al., 2016), can benefit from this. Diffuse hydrothermal sources are also essential contributors of iron to the deep waters (Le Bris et al., 2019; Lough et al., 2019b), and a similar small-scale profiling study and size fractionation analyses are needed to explore the distinct, possibly biologically transformed unique (nano)particle dynamics in and around vents.

**Figure 7:**
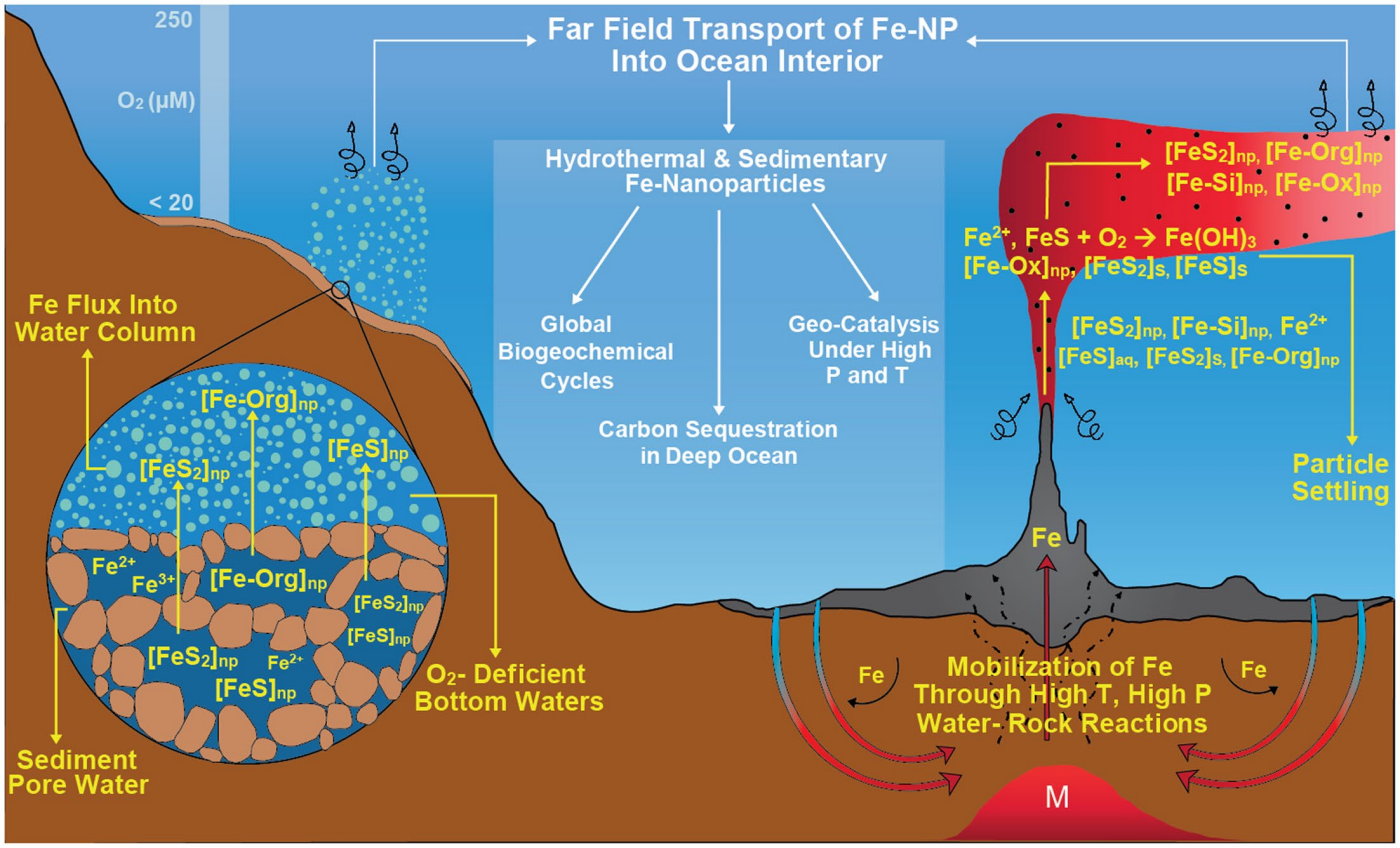
Nanoparticles as an important geochemical actor across naturally occurring marine redox gradients such as high-temperature hydrothermal vents and reducing sediments where metal turnover is orders of magnitude higher compared to other ocean zones. These nanoparticle fluxes might have a primary role in global biogeochemical cycles, carbon sequestration in deep ocean and geo-catalysis under high pressure and temperature conditions.

Finally, it must be emphasized that understanding such size fractionation and nanoparticle dynamics around vents is vital and not just from the traditional chemical oceanographic perspective that focuses on euphotic zone productivity. Iron speciation and size fractionation, through the high catalytic role of nanoparticles, should play a role in transforming deep-sea organic carbon pools and influence the biological interaction of metals and carbon. Global and regional models can therefore benefit from a better parametrization of iron fluxes, not only from vents, but also other systems such as seeps and reducing sediments. This will be necessary to eliminate uncertainties with regard to carbon cycling and enable better estimations of future climate states as well as improve our understanding of past climate shifts (glacial times, carbon sinks, etc.). The Earth system hosts many seafloor redox gradients, high-T, and diffuse vents to cold seeps and reducing sediments, in most of which filter-passing nanoparticles may be abundant across marine redox gradients where metal turnover is orders of magnitude higher than other ocean zones (Figure 7). These nanoparticle fluxes, in turn, could explain the persistence of benthic and hydrothermal iron fluxes in the ocean interior, as consistently reported in various oceanic transects arising from the GEOTRACES project (Resing et al., 2015; Fitzsimmons et al., 2017). Furthermore, nanoparticle and colloid formation and accumulation may amplify under redox oscillations frequently encountered in marine chemoclines (as a result of physical forcing) or in the mixing zone of reduced hot vents with the oxidized, cold seawater. This will gain importance as dramatic changes are anticipated in metal cycles: global warming, marine deoxygenation, and acidification may increase metal mobility in the Earth system, possibly amplifying internal metal fluxes between oceanic compartments (Basile-Doelsch et al., 2015; Eiriksdottir et al., 2015).

## Conclusion

We have observed that a colloidal fraction, cFe, is a persistent feature of hydrothermal vent mixing zone samples from the high-temperature source fluids at the Broken Spur (basalt-hosted) and Rainbow (serpentinite-hosted) vent fields of the Mid-Atlantic Ridge. The cFe fraction is either stable or generally grows with distance from the vent orifice, and our detailed analysis shows that it is a biogeochemically dynamic and significant pool. This colloidal pool changes in composition as the colloidal particles appear to be more HCl extractable in the samples more distant from the orifice. The HNO_3_ extractable fraction is significant in the mid-plumes in the sulfide-rich Broken Spur vents. Our results complement previous reports of dissolved Fe in MAR vents. With its rich geological variety of hydrothermal vents, nanoparticle export is a persistent feature of the Northern Mid-Atlantic Ridge. Moreover, we show for the first time that Broken Spur vents, with a different Fe:S end-member ratio, are also a principal source of nanoparticles to the near-field. We propose that this recalcitrant Fe pool – surviving the immediate precipitation – is the fraction that maintains high hydrothermal iron fluxes to the deep ocean. If vent iron inputs are shown to be sustained over different time periods, this previously overlooked mechanism has the capacity to sustain fluxes of ecosystem-limiting metals originating from the ocean’s interior.

## Data Availability Statement

The original contributions presented in the study are included in the article/Supplementary Material; further inquiries can be directed to the corresponding author.

## Author Contributions

MY and NLB designed the study and steered the sampling during the TRANSECT campaign. MY and SS performed the subsampling, onboard measurements, and sample fixation for on-shore analyses. MY, SS, and NLB analyzed the data and drafted the manuscript. All authors contributed to the article and approved the submitted version.

## Funding

The cruise was funded by the French Oceanographic Fleet and CNRS-INEE to LECOB (NLB). Transect Cruise DOI: https://doi.org/10.17600/18000513

## Conflict of Interest

The authors declare that the research was conducted in the absence of any commercial or financial relationships that could be construed as a potential conflict of interest.

## Publisher’s Note

All claims expressed in this article are solely those of the authors and do not necessarily represent those of their affiliated organizations, or those of the publisher, the editors and the reviewers. Any product that may be evaluated in this article, or claim that may be made by its manufacturer, is not guaranteed or endorsed by the publisher.
