# Supplementary material for: Soluble, Colloidal, and Particulate Iron Across the Hydrothermal Vent Mixing Zones in Broken Spur and Rainbow, Mid-Atlantic Ridge
Source: Front Microbiol. 2021 Oct 29;12:631885. doi: 10.3389/fmicb.2021.631885 (PMC8586216; doi:10.3389/fmicb.2021.631885)
Supplement: Supplementary file 1 [file Data_Sheet_1.docx]

Supplementary Material

# Supplementary Figures and Tables

## Supplementary Figures


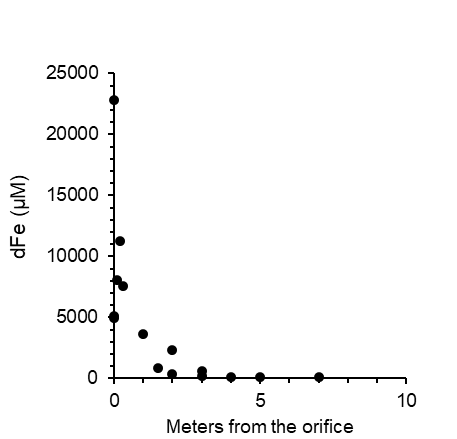

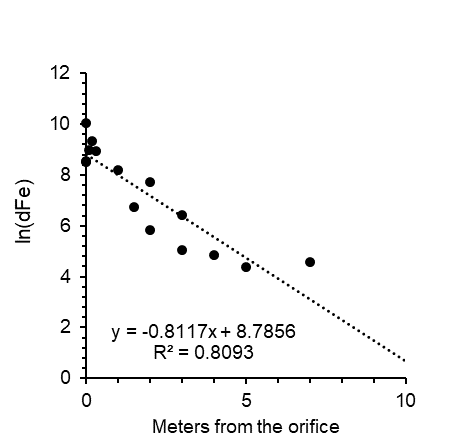

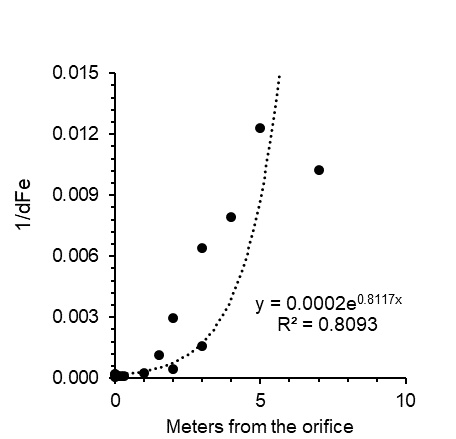


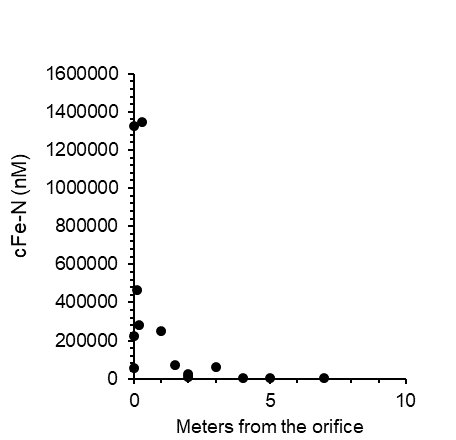

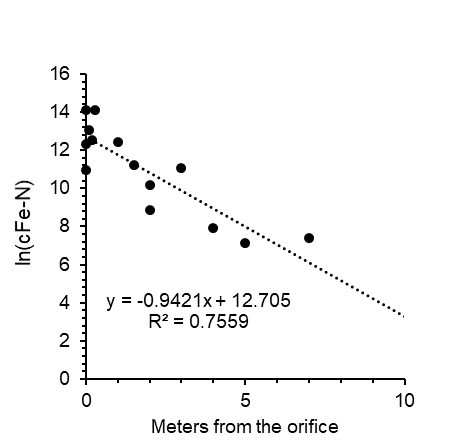

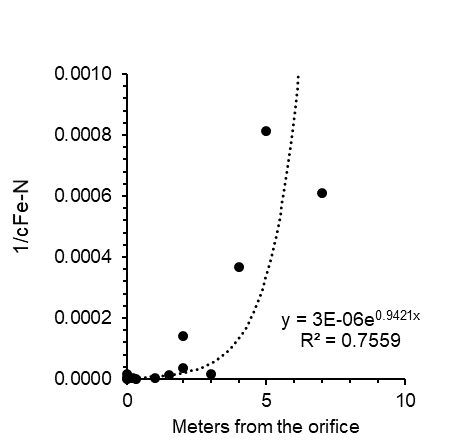


**Supplementary Figure S1.** Plots showing e-folding loss rates for dFe and cFe-N in Rainbow hydrothermal plume. Linear relationship observing in natural logarithm of dFe and cFe-N with respect to distance from orifice stating that apparent loss of dissolved iron phases is related with first order dependence of dFe and cFe-N concentrations.


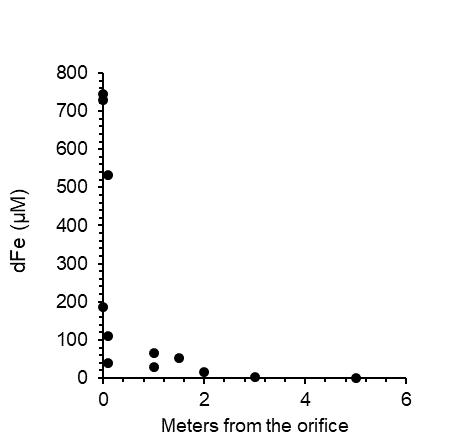

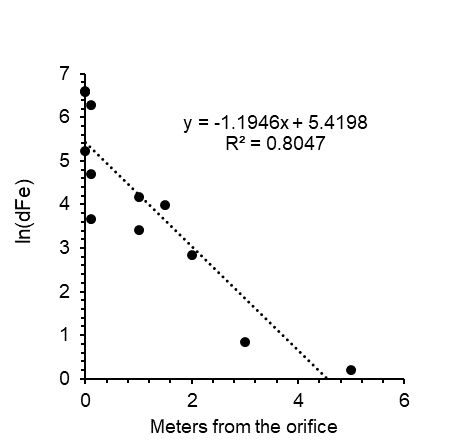

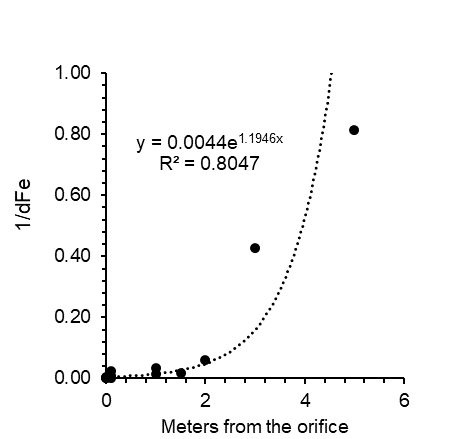


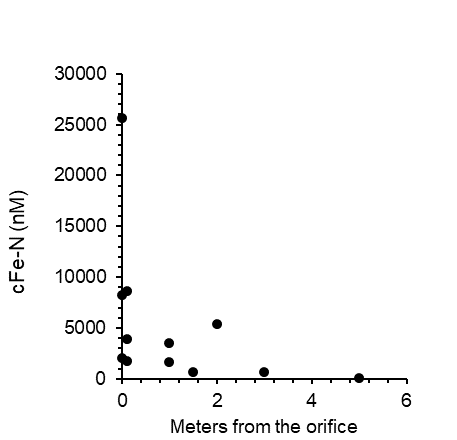

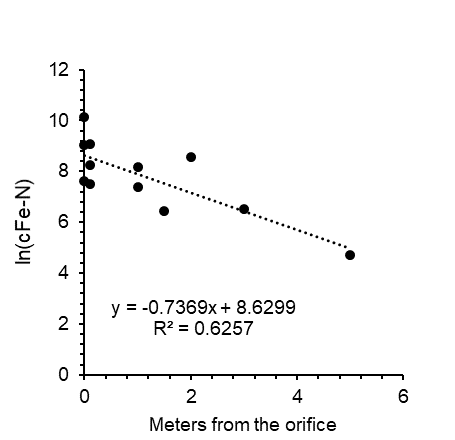

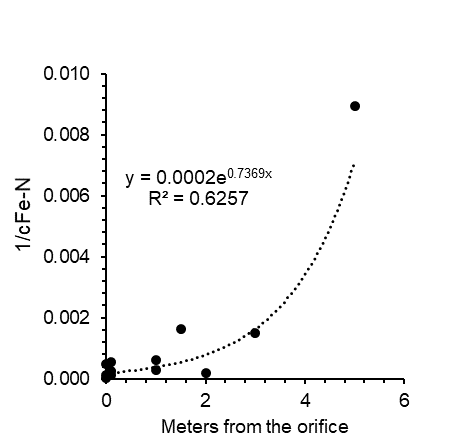


**Supplementary Figure S2.** Plots showing e-folding loss rates for dFe and cFe-N in Broken Spur hydrothermal plume. Linear relationship observing in natural logarithm of dFe and cFe-N with respect to distance from orifice stating that apparent loss of dissolved iron phases is related with first order dependence of dFe and cFe-N concentrations.

## Supplementary Tables

**Supplementary Table S1.** Table showing concentrations of dissolved iron (dFe), nitric acid-leachable nanoparticle colloids (cFe-N), soluble iron (sFe), colloidal iron (cFe), particulate iron (pFe), manganese (Mn), silica (Si), magnesium (Mg) and distances where samples were taken in Rainbow and Broken Spur hydrothermal plumes (FTD: Fumeur Tres Dense; FN: Fumeur Nouveau; n.m.: not measured).

| **Vent Field** | **Sampling (m)** | **dFe (µM)** | **cFe-N (µM)** | **sFe (µM)** | **cFe (µM)** | **pFe (µM)** | **Mn (µmol/kg)** | **Si (µmol/kg)** | **Mg (mmol/kg)** |
| --- | --- | --- | --- | --- | --- | --- | --- | --- | --- |
| **Rainbow** |  |  |  |  |  |  |  |  |  |
| FTD | 0 | 5112.5 | 224.1 | n.m. | n.m. | 318.2 | 392.0 | 1522.7 | 42.8 |
|  | 0.3 | 7601.9 | 1346.4 | n.m. | n.m. | 630.8 | 384.7 | 1506.9 | 38.8 |
|  | 2 | 337.3 | 7.1 | n.m. | n.m. | 173.1 | 37.7 | 340.7 | 51.5 |
|  | 3 | 155.9 | 0.2 | n.m. | n.m. | 131.3 | 22.6 | 296.8 | 49.6 |
|  | 5 | 0.0 | 0.0 | n.m. | n.m. | 104.0 | 33.3 | 345.0 | 31.4 |
|  | 12 | 0.0 | 0.0 | n.m. | n.m. | 28.9 | 3.0 | 153.3 | 46.0 |
| FN | 0 | 4964.8 | 57.6 | n.m. | n.m. | 13.7 | 208.7 | 1002.0 | 39.2 |
|  | 0.2 | 11294.0 | 281.3 | n.m. | n.m. | 0.0 | 737.0 | 3922.5 | 29.2 |
|  | 1.5 | 857.8 | 74.4 | n.m. | n.m. | 153.9 | 75.0 | 489.8 | 51.7 |
|  | 4 | 125.8 | 2.7 | n.m. | n.m. | 100.6 | 17.6 | 386.4 | 50.9 |
|  | 7 | 97.6 | 1.6 | n.m. | n.m. | 35.9 | 10.9 | 807.7 | 49.5 |
| Magali | 0 | 22791.3 | 1326.4 | 20642.9 | 2148.4 | 906.0 | 1397.7 | 6670.4 | 7.4 |
|  | 0.1 | 8070.3 | 467.0 | 7145.6 | 924.7 | 357.7 | 475.3 | 2621.5 | 29.1 |
|  | 1 | 3649.4 | 250.6 | 3383.8 | 265.5 | 94.5 | 237.6 | 651.4 | 39.1 |
|  | 2 | 2293.5 | 26.7 | 2225.0 | 68.6 | 117.8 | 137.3 | 827.7 | 40.5 |
|  | 3 | 625.1 | 62.6 | 542.0 | 83.1 | 133.7 | 45.7 | 310.5 | 45.6 |
|  | 5 | 81.2 | 1.2 | 68.9 | 12.3 | 50.0 | 12.7 | 574.8 | 48.2 |
| **Broken Spur** |  |  |  |  |  |  |  |  |  |
| Spire | 0 | 187.2 | 2.1 | 184.6 | 2.7 | 217.8 | 151.3 | 3536.4 | 24.1 |
|  | 0.1 | 39.5 | 1.8 | 36.4 | 3.1 | 27.0 | 31.2 | 738.6 | 46.3 |
|  | 1 | 30.1 | 1.6 | 24.0 | 6.1 | 13.6 | 14.9 | 610.1 | 42.9 |
|  | 2 | 17.0 | 5.3 | 11.9 | 5.1 | 12.9 | 15.2 | 566.9 | 40.7 |
|  | 5 | 1.2 | 0.1 | 0.8 | 0.4 | 1.6 | 10.1 | 565.4 | 49.6 |
| Chandelier | 0 | 743.6 | 8.2 | 707.7 | 35.9 | 82.6 | 267.3 | 8992.0 | 9.2 |
|  | 0.1 | 531.4 | 8.6 | 514.1 | 17.2 | 138.2 | 187.8 | 7751.5 | 18.7 |
|  | 1 | 65.2 | 3.5 | 39.8 | 25.4 | 8.4 | 19.4 | 1364.5 | 44.2 |
|  | 3 | 2.3 | 0.7 | 0.0 | 2.3 | 2.0 | 10.3 | 261.7 | 43.2 |
| Dragon | 0 | 728.7 | 25.6 | 569.5 | 159.1 | 19.1 | 330.4 | 6214.5 | 6.9 |
|  | 0.1 | 110.7 | 3.9 | 91.5 | 19.3 | 207.3 | 179.2 | 7842.4 | 22.9 |
|  | 1.5 | 53.5 | 0.6 | 47.2 | 6.4 | 19.9 | 21.3 | 2089.9 | 48.0 |
